# A Durable Response in Sinonasal Undifferentiated Carcinoma Treated With Subtotal Resection and Ultra-Early Postoperative Chemoradiotherapy: A Case Report

**DOI:** 10.7759/cureus.99738

**Published:** 2025-12-20

**Authors:** Kota Hiraoka, Shingo Umemoto, Kaori Tateyama, Takashi Hirano

**Affiliations:** 1 Department of Otorhinolaryngology - Head and Neck Surgery, Faculty of Medicine, Oita University, Yufu, JPN

**Keywords:** cisplatin-based chemotherapies, concurrent chemoradiotherapy (ccrt), early postoperative radiotherapy, sinonasal undifferentiated carcinoma, subtotal resection

## Abstract

Sinonasal undifferentiated carcinoma (SNUC) is a rare and aggressive malignancy that arises in the nasal cavity and paranasal sinuses. Although gross total resection (GTR) is associated with improved local control and survival, the anatomical complexity and frequent skull base invasion often render complete resection infeasible. In such cases, the timing of postoperative therapy may be critical for disease control. We report a case of a 68-year-old man with stage IV SNUC who underwent subtotal endoscopic resection, followed by ultra-early postoperative concurrent chemoradiotherapy (CCRT) initiated within one week of surgery. CCRT was delivered using intensity-modulated radiation therapy (IMRT) and cisplatin (CDDP). The patient achieved a complete response with no evidence of recurrence at six-year follow-up. This case underscores the potential for durable disease control through timely multimodal therapy in anatomically unresectable cases where GTR is not feasible. While GTR remains the optimal goal when achievable, early postoperative chemoradiotherapy may offer meaningful benefit in selected patients with subtotal resection. We discuss the significance of treatment timing, surgical extent, and multidisciplinary planning in the context of recent literature.

## Introduction

Sinonasal undifferentiated carcinoma (SNUC) is a highly aggressive and poorly differentiated epithelial malignancy, first reported by Frierson et al. in 1986 [1]. SNUC is a high-grade epithelial malignancy characterized by rapid local invasion, frequent skull base or orbital involvement, and a poor prognosis [2,3]. No universally established standard of care is available owing to its rarity and aggressive behavior. Historically, trimodal approaches involving surgery, radiation therapy (RT), and chemotherapy have been used; however, treatment sequencing remains controversial [4]. Although gross total resection (GTR) has traditionally been associated with improved outcomes, it is often unachievable in patients with advanced-stage disease [5]. The optimal treatment strategy for patients who cannot achieve GTR remains an important clinical question. Xu et al. reported that nearly 80% of SNUC cases are diagnosed at the T3 or T4 stage, underscoring the frequent anatomical limitations in achieving GTR [2]. In this context, the timing of postoperative therapy may be especially important for residual disease control. However, there is limited clinical evidence on the impact of ultra-early postoperative concurrent chemoradiotherapy (CCRT) (within one week of surgery) in patients with SNUC. We report a rare case in which the timely initiation of adjuvant CCRT following subtotal resection resulted in a durable complete response lasting six years.

## Case presentation

Initial presentation and clinical findings

**Figure 1:**
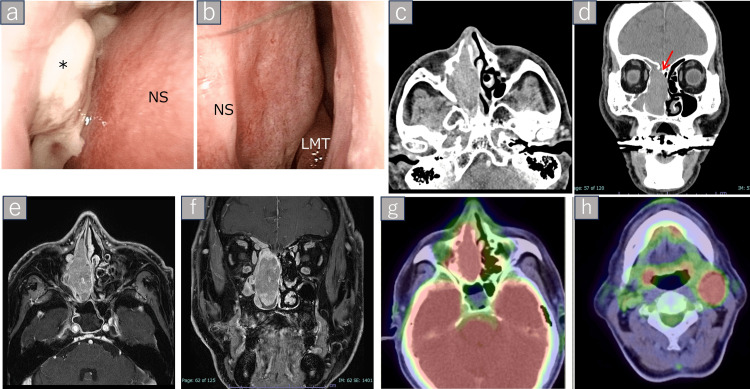
Preoperative endoscopic and imaging findings. The tumor is indicated by an asterisk (*) symbol. Endoscopic views of the nasal cavity. (a) Right nasal cavity showing a friable, hemorrhagic tumor occupying the nasal passage. (b) Left nasal cavity without evidence of tumor involvement. (b) Axial and (c) coronal CT images demonstrating bony erosion of the cribriform plate (arrow) and ethmoid sinuses. (d) Axial and (e) coronal contrast-enhanced MRI showing a heterogeneously enhancing mass extending from the nasal cavity to the anterior cranial base. (f) Axial and (g) coronal FDG-PET/CT demonstrating intense metabolic uptake in the primary tumor and left upper deep cervical lymph node, consistent with nodal metastasis. NS: nasal septum; LMT: left middle turbinate; FDG-PET: fluorodeoxyglucose-positron emission tomography

A 68-year-old man presented with epistaxis, left periorbital swelling, nasal obstruction, and headache, all of which had rapidly progressed over several weeks. Nasal endoscopy revealed a friable mass occupying the left nasal cavity (Figure 1, panels a, b). Neurologic examination showed no deficits. The patient denied vision loss or diplopia but reported increasing pressure around the right eye. Histopathological evaluation of a nasal biopsy confirmed a diagnosis of sinonasal undifferentiated carcinoma (SNUC).

Imaging findings

Contrast-enhanced computed tomography (CT) revealed a soft tissue mass occupying the right nasal cavity and ethmoid sinuses with bone erosion involving the ethmoid complex and cribriform plate (Figure 1, panels c, d). Subsequent contrast-enhanced magnetic resonance imaging (MRI) revealed a heterogeneously enhancing lesion extending to the anterior cranial base and the ethmoid plate (Figure 1, panels e, f). Fluorodeoxyglucose-positron emission tomography (FDG-PET)/CT demonstrated intense uptake in the primary tumor, as well as in a suspicious lymph node located at level IIa in the left upper deep cervical region (Figure 1, panels g, h).

Surgical findings

The surgical plan was to attempt gross total resection (GTR) if dissection along the anterior skull base appeared technically feasible. Preoperative imaging suggested probable invasion into the skull base, and subtotal resection was anticipated as a fallback option in the event of unfavorable intraoperative findings. Transcranial resection with skull base reconstruction was not planned, as the potential morbidity was considered to outweigh the benefits. Regardless of resection extent, the likelihood of a close margin at the skull base was recognized, and ultra-early initiation of concurrent chemoradiotherapy (CCRT) was prescheduled as part of the treatment strategy.

**Figure 2:**
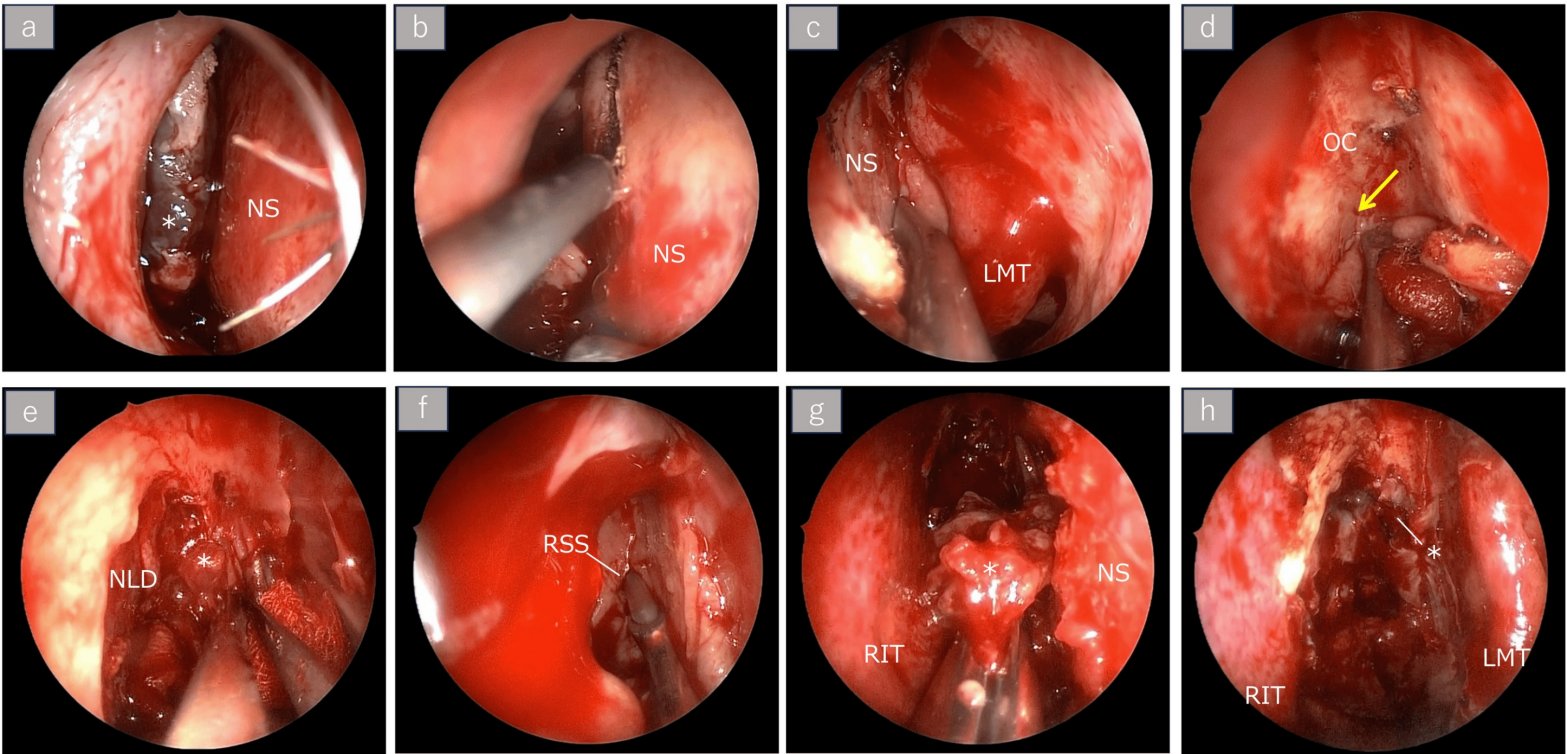
Intranasal operative findings. The tumor is indicated by an asterisk (*) symbol. Multiangle endoscopic views during subtotal resection demonstrating tumor extent, dissection landmarks, and residual disease. (a) Tumor occupying the right nasal cavity. (b) Mucosal incision was made anterior to the tumor in the right nasal cavity. (c) A corresponding incision was made on the left nasal septum to allow bilateral access. The resection margins of the nasal septum extended approximately 12 mm from the anterior septal edge, superiorly to the nasal roof, inferiorly to the nasal floor, and posteriorly to the anterior face of the sphenoid sinus. (d) Dissection advanced until the first olfactory nerve (arrow) was identified. (e) The tumor extended from the olfactory cleft to the anterior cranial base. Due to its hemorrhagic nature, resection was performed near the cranial base. (f) Tumor involvement was noted in the posterior ethmoid sinus roof, with purulent discharge from the sphenoid sinus. (g) Tumor resected in multiple fragments. (h) Final operative field showing residual tumor at the olfactory cleft and anterior cranial base. No preventive measures were taken to avoid septal perforation, as tumor clearance was prioritized. NS: nasal septum; LMT: left middle turbinate; OC: olfactory cleft; NLD: nasolacrimal duct; RSS: right sphenoid sinus; RIT: right inferior turbinate

Right nasoparanasal sinus tumor resection and left cervical lymph node dissection were performed under general anesthesia. The middle turbinate was displaced by the tumor (Figure 2, panel a). For the right nasal cavity tumor, a mucosal flap was elevated from the nasal septum to expand the surgical field (Figure 2, panel b). A portion of the nasal septum was resected to enable the manipulation of both nasal cavities (Figure 2, panel c). The extent of septal mucosal resection included approximately 12 mm from the anterior nasal septal edge, extending superiorly to the roof line, inferiorly to the nasal floor, and posteriorly to the anterior face of the sphenoid sinus, preserving the posterior septum. No specific measures were taken to prevent residual septal perforation, as tumor clearance was prioritized. Dissection was carried out in a continuous manner from the lateral nasal cavity to the nasal septal mucosa, progressing toward the olfactory cleft and reaching the level of the first olfactory thread (Figure 2, panel d). The tumor had infiltrated the anterior cranial base and was highly hemorrhagic; therefore, complete resection was deemed difficult, resulting in a fragmented resection (Figure 2, panels e-g). Thus, although a portion of the tumor remained in the anterior cranial base, >95% tumor reduction was achieved (Figure 2, panel h).

**Figure 3:**
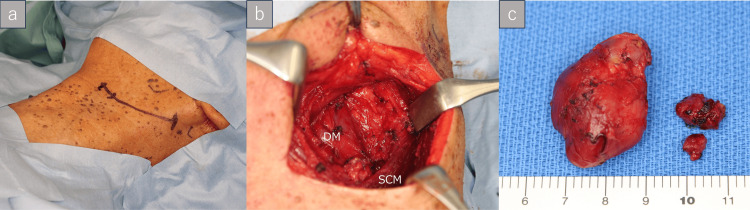
Surgical findings of left neck dissection. (a) A planned left upper horizontal cervical incision along the natural skin crease. (b) Intraoperative view following completion of neck dissection. (c) Resected cervical lymph node specimens. SCM: sternocleidomastoid muscle; DM: digastric muscle

Although preoperative imaging suggested possible contralateral nodal involvement, no pathological confirmation of metastasis had been established prior to surgery. Therefore, a left upper neck dissection was performed for both diagnostic and therapeutic purposes, with removal of the enlarged lymph node and surrounding nodes in level IIa (Figure 3, panels a-c).

Pathological findings

**Figure 4:**
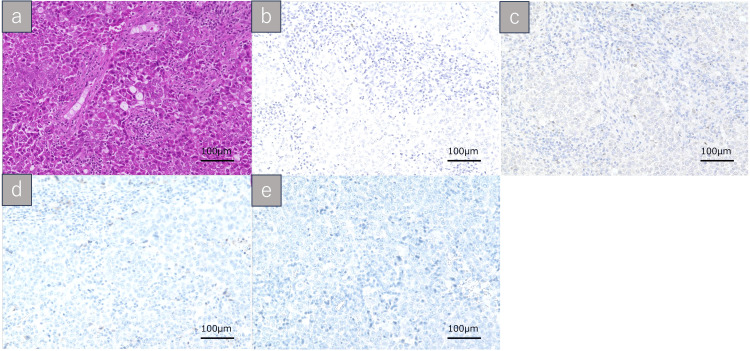
Histopathological findings of the nasal tumor specimen. (a) Hematoxylin and eosin staining reveals nests of poorly differentiated tumor cells with vesicular nuclei, prominent nucleoli, and amphophilic cytoplasm, arranged in a focally alveolar pattern. (b-e) Immunohistochemical findings: (b) EBER-ISH, (c) p63, (d) CK5/6, and (e) p40 are all negative. These results support the diagnosis of sinonasal undifferentiated carcinoma (SNUC). All images were captured at 200× magnification. Scale bar: 100 μm. EBER-ISH: EBER in situ hybridization

Histopathological examination of the nasal cavity specimens revealed nests of markedly atypical cells with vesicular nuclei, prominent nucleoli, and amphophilic cytoplasm. The tumor exhibited a focal alveolar growth pattern (Figure 4, panel a). Focal areas of necrosis were present, without evidence of keratinization. Immunohistochemical staining was negative for CK5/6, p63, p40, and EBER (in situ hybridization) (Figure 4, panels b-e). These findings supported the diagnosis of an undifferentiated sinonasal carcinoma. Margin assessment was inconclusive owing to the fragmented nature of the resected specimen. Metastasis was confirmed in a 25-mm left cervical lymph node located at level IIa, without extranodal extension or lymphovascular invasion. The final pathological stage was pT3N2cM0 (stage IVa).

Postoperative course

**Figure 5:**
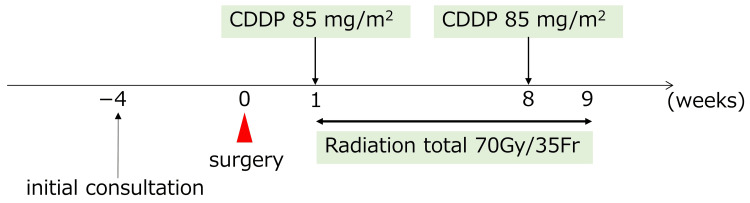
Clinical course and treatment timeline. Surgical resection was performed four weeks after the initial consultation. Concurrent chemoradiotherapy was initiated one week postoperatively and consisted of radiation therapy (RT) and cisplatin (CDDP). CDDP was administered at a dose of 85 mg/m² per cycle for two cycles. CDDP: cisplatin

**Figure 6:**
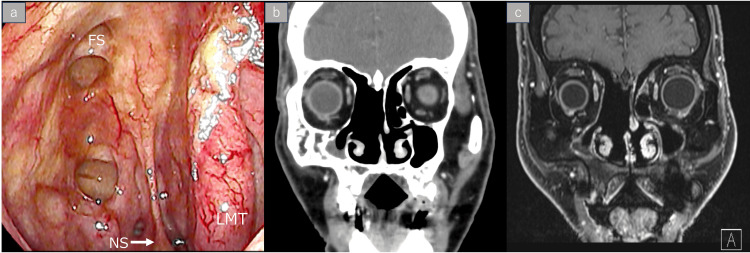
Disease status at six-year follow-up. Endoscopic and imaging findings demonstrated no evidence of disease recurrence six years after treatment. A residual nasal septal perforation, likely attributable to surgical and chemoradiation effects, was observed. (a) Nasal endoscopy showing a residual septal perforation. (b) Coronal contrast-enhanced CT. (c) Coronal contrast-enhanced MRI. NS: nasal septum; LMT: left middle turbinate; FS: frontal cell

Concurrent chemoradiotherapy (CCRT) was initiated one week postoperatively, and radiation therapy (RT) was delivered at a total dose of 70 Gy in 35 fractions with concurrent cisplatin (CDDP) (85 mg/m^2^, q3w, two cycles) adjusted for tolerability (Figure 5). No major complications occurred, and the patient completed the treatment without delay. Complete remission was confirmed on imaging and endoscopy, with no evidence of recurrence at six-year follow-up (Figure 6, panels a-c). On follow-up endoscopy, a residual septal perforation was noted, likely due to the combined effects of surgical manipulation and radiation therapy. The patient remained asymptomatic from this finding throughout the follow-up period.

## Discussion

Surgical extent and GTR limitations

Gross total resection (GTR) is traditionally considered the gold standard in SNUC due to its association with improved local control and survival [4-6]. However, GTR is often not feasible in T3-T4 tumors because of anatomical constraints, such as skull base or orbital invasion [2].

Subtotal resection followed by early chemoradiotherapy

We performed a subtotal resection to avoid a delay in chemoradiotherapy, which might have resulted from extensive skull base resection and reconstruction. Abdelmeguid et al. emphasized the importance of minimizing delays in postoperative CCRT; postponement of treatment may compromise disease control because of the aggressive biology of SNUC [7]. Similarly, Christopherson et al. highlighted the significance of minimizing treatment delays to maximize local control [5].

Subtotal resection in other sinonasal malignancies

This approach may be conceptually supported by findings in other sinonasal malignancies. For instance, Klymenko et al. reported that multimodal treatment, including surgery and adjuvant chemoradiotherapy with advanced techniques such as intensity-modulated radiation therapy (IMRT), was associated with favorable outcomes in squamous cell carcinoma (SCC) and esthesioneuroblastoma. Although the study did not specifically compare gross total vs. subtotal resection, their results suggest that effective postoperative therapy may contribute to disease control even in anatomically challenging cases [8]. In addition, the EA3163 trial for T3-T4a nasal and paranasal sinus SCC supports organ-preserving approaches coupled with effective chemoradiation [9].

Trimodality vs. CCRT alone

A review of the National Cancer Database concluded that although trimodality therapy provides the best survival outcomes, CCRT alone represents a reasonable alternative when surgery is not feasible or must be limited in extent [10]. These findings support flexible and patient-centered treatment planning. Similarly, Kono et al. reported a case of unresectable T4b SNUC that was successfully treated with definitive platinum-etoposide CCRT, using RT with a simultaneous integrated boost [11]. In their report, the patient achieved complete remission and visual function recovery. Although our case involved a surgical approach followed by early CCRT, this report emphasizes that nonsurgical curative strategies remain a viable alternative in carefully selected patients.

Chemotherapy considerations and dose modification

Our patient received CDDP at a reduced dose (85% of the standard dose) owing to concerns regarding compliance and tolerability. Despite this reduction, the patient developed grade 2 neutropenia (Common Terminology Criteria for Adverse Event {CTCAE} v5.0), suggesting that full-dose administration might have necessitated treatment interruption. Although the total cumulative dose did not reach the conventional threshold (e.g., 200 mg/m^2^), previous studies have demonstrated that reduced-dose CDDP may retain therapeutic efficacy, particularly in combination with high-dose RT. Bauml et al. and Szturz et al. emphasized the need for individualized decision-making in CDDP use, balancing toxicity risk with treatment benefit, particularly in patients at risk for renal, auditory, or general performance-related toxicities [12,13]. Yoshida et al. emphasized the importance of individualized treatment strategies for patients undergoing multimodal therapy [4].

Induction chemotherapy

Induction chemotherapy (IC) was not administered in this case, as its role in SNUC remains controversial. Although IC has been explored in several studies, its efficacy is inconclusive. Burggraf et al. noted that while IC may assist in identifying responders suitable for nonsurgical management, no consistent survival benefit has been demonstrated [14]. Furthermore, Papazian et al. reported a case in which IC failed to achieve a response, and the patient experienced early recurrence despite subsequent GTR and adjuvant chemoradiotherapy [15]. In the present case, GTR was considered potentially feasible based on imaging, but skull base invasion raised uncertainty. Therefore, we prioritized early definitive local therapy with subtotal resection and ultra-early postoperative CCRT, aiming to minimize treatment delays and address the anticipated close margin at the skull base.

Molecular profiling and immune checkpoint inhibition

Recent advances in molecular profiling have uncovered potentially actionable targets for sinonasal malignancies, including SNUC. Jakimovska et al. highlighted recurrent SMARCB1 loss and PD-L1 expression as promising biomarkers of SNUC [16]. Although immune checkpoint inhibitors are not yet considered standards in this setting, these findings support further investigation of immunotherapeutic approaches, particularly for unresectable or refractory diseases. Hoke et al. have emphasized the growing role of genomics in guiding future treatment strategies [17].

Role of particle therapy

Proton therapy is a compelling modality for sinonasal tumors that require high-dose conformal irradiation while sparing adjacent critical structures. Dagan et al. reported favorable local control and survival outcomes in patients with sinonasal malignancies, including SNUC, treated with proton therapy. They demonstrated that particle therapy can be tailored to tumor biology and resection status by incorporating approaches, such as accelerated fractionation. Treatment regimens, such as 1.2 Gy twice daily for a total of 66 Gy, were employed to select high-risk or incompletely resected tumors and counteract their rapid repopulation, thereby reducing treatment time without compromising safety [18].

This flexibility in dose and fractionation, along with precise dose delivery, underscores the utility of particle therapy in anatomically complex and aggressive sinonasal cancers. Further studies are warranted to define the optimal indications and regimens, particularly for patients who are unable to achieve gross total resection.

Although postoperative chemoradiotherapy is commonly initiated within four to six weeks to allow for wound healing, recent literature emphasizes the importance of minimizing delay, particularly in aggressive tumors such as SNUC [7]. In the present case, rapid postoperative recovery and the high-risk nature of the residual disease led us to begin CCRT on postoperative day seven. This approach warrants further exploration in selected patients, balancing the potential benefits of early intervention against considerations of wound healing. This discussion is summarized in Table 1.

**Table 1:** Overview of key discussion points and associated literature. GTR: gross total resection; CCRT: concurrent chemoradiotherapy; SCC: squamous cell carcinoma; IC: induction chemotherapy; IMRT: intensity-modulated radiation therapy

Treatment modality	Key insights	Supporting references
Surgical extent	GTR associated with improved outcomes, but often unachievable in T3-T4 disease.	[2,5,6]
Subtotal resection + early CCRT	Subtotal resection followed by prompt CCRT can yield favorable outcomes.	[5,7]
Other sinonasal malignancies	Subtotal resection + CCRT is effective in SCC and esthesioneuroblastoma.	[8,9]
Trimodality vs. CCRT alone	Trimodality best survival; CCRT alone viable when surgery is limited.	[10,11]
Cisplatin dose modification	Reduced dose may retain efficacy; individualization is key.	[4,12,13]
Induction chemotherapy	IC may help stratify patients, but benefit remains inconclusive; failure may limit options.	[14,15]
Molecular profiling and immune checkpoint inhibition	Molecular targets identified; immunotherapy under investigation.	[16,17]
Particle therapy	Proton therapy beneficial for residual disease and complex anatomy.	[18]

## Conclusions

This case illustrates the potential for durable control of locally advanced SNUC through subtotal resection followed by early CCRT. While GTR remains the standard goal when technically and safely achievable, subtotal resection may represent a pragmatic alternative in anatomically unresectable cases or when GTR would incur excessive morbidity or delay adjuvant treatment. In such settings, timely initiation of postoperative CCRT is critical, and the integration of advanced radiotherapy and chemotherapy strategies enables personalized care tailored to individual patient and disease characteristics.

## References

[1] Frierson Jr HF, Mills SE, Fechner RE, Taxy JB, Levine PA (1986). Sinonasal undifferentiated carcinoma: an aggressive neoplasm derived from schneiderian epithelium and distinct from olfactory neuroblastoma. Am J Surg Pathol.

[2] Xu CC, Dziegielewski PT, McGaw WT, Seikaly H (2013). Sinonasal undifferentiated carcinoma (SNUC): the Alberta experience and literature review. J Otolaryngol Head Neck Surg.

[3] Resteghini C, Castelnuovo P, Nicolai P (2023). The SINTART 1 study. A phase II non-randomised controlled trial of induction chemotherapy, surgery, photon-, proton- and carbon ion-based radiotherapy integration in patients with locally advanced resectable sinonasal tumours. Eur J Cancer.

[4] Yoshida E, Aouad R, Fragoso R, Farwell DG, Gandour-Edwards R, Donald PJ, Chen AM (2013). Improved clinical outcomes with multi-modality therapy for sinonasal undifferentiated carcinoma of the head and neck. Am J Otolaryngol.

[5] Christopherson K, Werning JW, Malyapa RS, Morris CG, Mendenhall WM (2014). Radiotherapy for sinonasal undifferentiated carcinoma. Am J Otolaryngol.

[6] Chen AM, Daly ME, El-Sayed I, Garcia J, Lee NY, Bucci MK, Kaplan MJ (2008). Patterns of failure after combined-modality approaches incorporating radiotherapy for sinonasal undifferentiated carcinoma of the head and neck. Int J Radiat Oncol Biol Phys.

[7] Abdelmeguid AS, Bell D, Hanna EY (2019). Sinonasal undifferentiated carcinoma. Curr Oncol Rep.

[8] Klymenko O, Buchberger AM, Wollenberg B (2021). Radiooncological view on therapy outcome after multidisciplinary treatment of sinonasal tumors. Cancers (Basel).

[9] Saba NF, Flamand Y, Lin DT (2025). Neoadjuvant chemotherapy and surgery versus surgery for organ preservation of T3 and T4a nasal and paranasal sinus squamous cell carcinoma: ECOG-ACRIN EA3163. Clin Cancer Res.

[10] Lillo S, Mirandola A, Vai A (2024). Current status and future directions of proton therapy for head and neck carcinoma. Cancers (Basel).

[11] Kono S, Ando R, Nakamizo M, Nagashima Y, Hashimoto Y (2025). Successful management of advanced sinonasal undifferentiated carcinoma using concurrent chemoradiotherapy with simultaneous integrated boost and intensity-modulated radiation therapy: a case report. Cureus.

[12] Bauml JM, Vinnakota R, Park YH (2019). Cisplatin every 3 weeks versus weekly with definitive concurrent radiotherapy for squamous cell carcinoma of the head and neck. J Natl Cancer Inst.

[13] Szturz P, Wouters K, Kiyota N (2019). Low-dose vs. high-dose cisplatin: lessons learned from 59 chemoradiotherapy trials in head and neck cancer. Front Oncol.

[14] Burggraf M, Schiele S, Thölken R, López FJ, Elawany N, Zenk J, Doescher J (2024). Contemporary treatment and outcome of sinonasal undifferentiated carcinoma: a meta-analysis. Head Neck.

[15] Papazian MR, Gordon AJ, Chow M, Patel A, Pacione D, Lieberman S, Givi B (2022). Sinonasal undifferentiated carcinoma with failed response to induction chemotherapy. J Neurol Surg Rep.

[16] Jakimovska F, Stojkovski I, Kjosevska E (2024). Nasal cavity and paranasal sinus cancer: diagnosis and treatment. Curr Oncol Rep.

[17] Hoke AT, Takahashi Y, Padget MR (2024). Targeting sinonasal undifferentiated carcinoma with a combinatory immunotherapy approach. Transl Oncol.

[18] Dagan R, Uezono H, Bryant C, Holtzman AL, Morris CG, Mendenhall WM (2021). Long-term outcomes from proton therapy for sinonasal cancers. Int J Part Ther.

